# Study of the Correlation Between Multi-parametric MRI (MP-MRI) Prostate Findings and Transrectal Ultrasound (TRUS)-Guided Prostate Biopsy Results in Patients With Raised Serum PSA

**DOI:** 10.7759/cureus.86216

**Published:** 2025-06-17

**Authors:** Deepak Sharma, Devpriya Mitra, Vikas K Bansal, Kunwar V Singh

**Affiliations:** 1 Urology, Shalby Sanar International Hospital, Gurgaon, IND; 2 Urology, Marengo Asia Hospital, Faridabad, IND; 3 Urology, Yashoda Hospital and Research Centre, Ghaziabad, IND; 4 Urology, Pushpawati Singhania Research Institute (PSRI) Hospital, New Delhi, IND

**Keywords:** gleason's score, mp mri prostate, pirads score, prostate cancer, trus guided biopsy

## Abstract

Background

Prostate cancer requires early and accurate diagnosis to improve outcomes of various treatment modalities. Multi-parametric MRI (MP-MRI) has emerged as a non-invasive imaging modality for detecting and evaluating prostate cancer for histological diagnosis; however, transrectal ultrasound (TRUS)-guided biopsy is still the gold standard.

Aim

To study the correlation between MP-MRI prostate findings using Prostate Imaging Reporting and Data System (PIRADS) scoring and TRUS-guided prostate biopsy results, including Gleason’s score, in patients with raised serum PSA.

Materials and methods

The present prospective research comprises 66 patients aged 40-80 years with PSA >4 ng/ml or free-to-total PSA ratio <0.15, who attended the urology department at Pushpawati Singhania Research Institute, New Delhi, between May 2018 and March 2020. MP-MRI was performed on all patients, along with cognitive MP-MRI-targeted biopsy and systematic 12-core TRUS-guided biopsy. Gleason’s scoring and immunohistochemistry (IHC) were used to confirm malignancy.

Results

MP-MRI revealed that 71.21% of patients had probably malignant or malignant findings (PIRADS 4/5). TRUS biopsy confirmed malignancy in 72.7% of patients. Gleason's score and the PIRADS score showed a strong connection (p <0.05). MP-MRI showed a 72.22% specificity, 87.5% sensitivity, 68.42% negative predictive value (NPV), 89.36% positive predictive value (PPV), and 83.33% diagnostic accuracy for detecting prostate cancer. Cognitive MP-MRI-targeted biopsy detected cancer in 66.67% of patients, while systematic biopsy detected it in 56%. The combination of both methods yielded the highest diagnostic accuracy. Inter-rater kappa agreement between MP-MRI and the TRUS biopsy was moderately strong (κ = 0.587, p<0.001).

Conclusion

MP-MRI has been a highly sensitive and specific modality in identifying clinically significant prostate cancer. PI-RADS scoring offers a standardized and reproducible method for evaluating prostate lesions. A cognitive MP-MRI-targeted TRUS biopsy, especially in conjunction with systematic biopsy, significantly improves prostate cancer detection rates, especially in PIRADS 4 or higher cases.

## Introduction

Prostate cancer is the most frequently diagnosed cancer in men, after lung cancer, worldwide [1]. Prostate cancer accounts for 7.4% of men's cancer fatalities worldwide, making it the sixth most prevalent cause of cancer-related mortality [2]. Over the last several decades, there has been a significant shift in the method utilized for identifying prostate carcinoma. Various conventional techniques like digital rectal examination, biochemical investigation in the form of serum prostate-specific antigen, and TRUS of the prostate are being employed. The recent addition of MP-MRI of the prostate has revolutionized the diagnosis of prostate carcinoma. It is being demonstrated that multiparametric MR imaging, which combines functional MR imaging methods like dynamic contrast-improved imaging, diffusion-weighted imaging, as well as apparent diffusion coefficient mapping, improves prostate cancer early detection, initial staging, and post-treatment follow-up [3]. Conventional MR imaging of prostate cancers results in ill-defined low signal intensity lesions on T2-weighted imaging, which might also be mimicked by a few benign conditions like prostatitis, hemorrhage, fibrosis, and post-intervention/treatment scarring [4]. Diffusion-weighted imaging depicts the microscopic Brownian motion of the water molecules within tissues in a magnetic field. These signals may be altered due to alterations in cellularity, cell membrane integrity, reduced extracellular space or fluid viscosity, which can be visualized as areas of restricted diffusion on diffusion-weighted imaging (high signal intensity) with corresponding low signal intensity on apparent diffusion coefficient (ADC) mapping. By quantifying the degree of diffusion, ADC maps offer a quantitative examination of DWI [5,6]. Dynamic contrast enhancement MRI is based on the principle of tumor angiogenesis, which is seen in any tumor of more than 2 mm in size [7]. When contrast is administered, enhanced tumor vascularity leads to an early enhancement compared to benign tissue. The conventional method for diagnosing prostate cancer is a random prostate biopsy guided by transrectal ultrasound (TRUS).

## Materials and methods

Present research has been performed on 66 patients who presented to the outpatient department and were admitted to the Department of Urology, Pushpawati Singhania Research Institute and Hospital, Sheikh Sarai, New Delhi, between May 2018 and March 2020.

Inclusion criteria

Age between 40 to 80 years with signed informed consent, PSA >4 ng/ml or free-to-total PSA ratio <0.15 (wherever clinically indicated), no evidence of PSA elevated by noncancerous factors, like urinary tract infection, bladder stones, or catheterization.

Exclusion criteria

Previous prostate biopsy or prostate surgery, known contraindication for MRI evaluation, patients with an associated renal dysfunction, or patients not willing to give consent.

The outcome of the study was measured in terms of the strength of the relationship between the Prostate Imaging Reporting and Data System (PIRADS) score on MP-MRI [3] and the Gleason‘s score result of TRUS-guided biopsy. Secondary outcomes were measured in the form of prostate cancer yield rate of systematic 12-core TRUS-guided prostate biopsy, with results of cognitive MP-MRI transrectal ultrasound targeted biopsy using the Philips MR Ingenia Prodiva 16-channel volume 1.5 T MRI scanner (Philips, Amsterdam, Netherlands).

TRUS-guided prostate biopsy

Preparation for the procedure included a cleansing enema prior to biopsy, and prophylactic antibiotics starting one day before. Additionally, 2% lignocaine jelly was administered as a local anesthetic before the biopsy. The patient was placed in the left lateral decubitus position with a flexed right knee and hip. Prostate biopsy was done by the 18-gauge, 25-cm long semi-automatic biopsy gun, introduced with the help of a biopsy guide fitted on the TRUS probe. Patients were subjected to a systematic 12-core TRUS-guided prostate biopsy irrespective of MP-MRI findings, along with a cognitive MP-MRI transrectal ultrasound targeted prostate biopsy under standard antibiotic cover. Each biopsy core was collected in a separate container filled with formalin, labeled, and sent for histopathological examination. The results were compared in the form of grades of the PIRADS score (Appendices) and positive TRUS biopsy results in the form of Gleason‘s score. Cognitive MP-MRI-targeted TRUS biopsy results were compared with systematic biopsy results. As per institute protocol, subjects who were showing atypical acinar cell proliferation on biopsy were further investigated with IHC using AMACR, p63, and HMWCK.

Statistical analysis

Statistical tests were applied to address the specific objectives of the study. Gleason's score and the MP-MRI PIRADS score were correlated by applying Spearman's rank correlation coefficient. To determine the strength of agreement between the MP-MRI PIRADS score and transrectal ultrasound biopsy results, inter-rater kappa agreement analysis was performed. Furthermore, diagnostic test analysis was conducted for assessing the negative predictive value (NPV), specificity, sensitivity, as well as positive predictive value (PPV) of MP-MRI in predicting malignancy. All analyses were deemed statistically significant if p<0.05. The Statistical Package for Social Sciences (SPSS), version 21.0 (IBM Corp., Armonk, NY, US), has been employed for statistical analysis after Microsoft Excel (Microsoft Corp., Redmond, WA, US) was leveraged for data input.

## Results

Table 1 depicts that approximately 40% (n=26) of the study subjects did not have any physical symptoms. Most of these patients presented to us with raised serum (S.) PSA found on routine health check-ups. Most symptomatic subjects had complaints of poor urinary flow and nocturia. Fifty percent (50%; n=33) of the study subjects were found to have normal digital rectal examination findings, and the rest of the subjects had either a suspicious nodule or induration present on physical examination.

**Table 1:** Basic demographic pattern and clinical features among patients

Category	Variable	No. of Patients/Value	Percentage/Description
Age Distribution	≤60 years	12	18.18%
	61–70 years	33	50.00%
	71–80 years	21	31.82%
	Mean ± SD	66.82 ± 6.9	–
	Median (IQR)	68.5 (62.25–72)	–
	Range	49–78	–
Symptom Distribution	Asymptomatic	26	39.39%
	Symptomatic	40	60.60%
	Total	66	100.00%
Complaints Among Symptomatic Subjects	Poor flow	32	48.48%
	Urgency	9	13.64%
	Frequency	11	16.67%
	Nocturia	26	39.39%
	Sense of incomplete voiding	15	22.72%
	Hematuria	1	1.52%
	Back pain	7	10.61%
Digital Rectal Examination Findings	Normal	33	50.00%
	Abnormal (nodule/induration)	33	50.00%
	Total	66	100.00%

Table 2 denotes the involvement of the peripheral zone and the transitional zone based on the kind of contribution and unilateral or bilateral involvement. Most of the study subjects had focal involvement in the peripheral zone and diffuse bilateral involvement in the transitional zone. Extra-prostatic extension is seen in the form of capsule involvement in 15% (n=10) of the subjects, neurovascular bundle involvement in 3% (n=2) subjects, seminal vesicle involvement in 17% (n=11), and lymph node involvement in 14% (n=9) of the subjects.

**Table 2:** MP-MRI and T2WI findings of study subjects MP-MRI: multi-parametric MRI; T2WI: T2-weighted imaging; PIRADS: Prostate Imaging Reporting and Data System

Category	Variable	No. of Subjects	Percentage
T2WI Findings – Peripheral Zone (PZ)	Diffuse involvement	14	21.21%
	Focal involvement	48	72.73%
	Normal	4	6.06%
Side of Peripheral Zone Involved (n=62)	Bilateral	32	51.61%
	Left lobe	15	24.19%
	Right lobe	15	24.19%
T2WI Findings – Transitional Zone (TZ)	Diffuse involvement	23	34.85%
	Focal involvement	11	16.67%
	Normal	32	48.48%
Side of Central Zone Involved (n=34)	Bilateral	30	88.24%
	Left	1	2.94%
	Right	3	8.82%
MP-MRI Findings	Diffusion-Weighted Imaging Changes	63	95.45%
	Apparent Diffusion Coefficient (ADC) Reversal	46	69.70%
	Capsule Involvement	10	15.15%
	Neurovascular Bundle Involvement	2	3.03%
	Seminal Vesicle Involvement	11	16.67%
	Lymph Node Involvement	9	13.64%
Dynamic Contrast Enhancement (Curve Type)	Type I	3	4.55%
	Type II	19	28.79%
	Type III	44	66.67%
PZ PIRADS Score	Score 2	4	6.15%
	Score 3	16	24.62%
	Score 4	32	49.23%
	Score 5	13	20.00%
TZ PIRADS Score	Score 2	10	33.33%
	Score 3	13	43.33%
	Score 4	5	16.67%
	Score 5	2	6.67%

Table 3 shows the distribution of the study subjects based on the PIRADS score obtained on MP-MRI. Approximately, 4.55% (n=3) were probably benign (PIRADS 2), 24.24% (n=16) of the study subjects were equivocal for the likelihood of the presence of cancer prostate (PIRADS 3), and approximately 71% (n=47) of the subjects were categorized into probably malignant/malignant category (PIRADS 4/5). Seventy-two point seven percent (72.7%; n=48) of the study subjects were finally diagnosed to have prostate cancer on TRUS biopsy, including cognitive MP-MRI targeted and systematic biopsy results. Two subjects were found to be positive on immunohistochemistry are included among the positive subjects. Fifty-three percent (53%; n=37) of the study subjects were found to have clinically significant prostate cancer. A total of 10 subjects out of the total reported cases were found to have atypical acinar cell proliferation on the biopsy report, who were further investigated with immunohistochemistry (IHC), and two of those patients were found to be positive and included among the subjects positive for prostate cancer.

**Table 3:** Diagnostic findings and biopsy results of study subjects MP-MRI: multi-parametric MRI; IHC: immunohistochemistry; PIRADS: Prostate Imaging Reporting and Data System

Category	Variable	No. of Subjects	Percentage
Final MP-MRI PIRADS Score	Score 2	3	4.55%
	Score 3	16	24.24%
	Score 4	33	50.00%
	Score 5	14	21.21%
MP-MRI Impression	Benign and equivocal	19	28.79%
	Probably malignant / Malignant	47	71.21%
Transrectal Ultrasound Biopsy Results	Benign	18	27.27%
	Malignant	48	72.73%
	Total	66	100.00%
Gleason’s Score (Among Malignant Cases)	Score 6	11	23.91%
	Score 7	11	23.91%
	Score 8	17	36.96%
	Score 9	7	15.22%
	Total (n = 46)		100.00%
IHC Results (In Atypical Acinar Cases)	IHC Negative	8	80.00%
	IHC Positive	2	20.00%
	Total (n = 10)		100.00%
Cognitive Targeted Biopsy Results	Negative	22	33.33%
	Positive	44	66.67%
	Total	66	100.00%
Systematic Biopsy Results	No	29	43.94%
	Yes	37	56.06%
	Total	66	100.00%

Out of 20 cases that did not turn out to be positive on TRUS biopsy, 10 patients were found to have atypical acinar cell proliferation and were subsequently investigated with immunohistochemistry with AMACR, p63, and HMWCK. Two patients of these were found to be positive for IHC, which signifies a high probability of clinically significant prostate cancer; hence, calculated as positive TRUS biopsy results for prostate cancer.

The above graph depicts the number of study subjects discovered to be positive for prostate cancer on cognitive MP-MRI-targeted TRUS biopsy. Sixty-six point sixty seven (66.67%; n=44) of the study subjects were ultimately demonstrated to be positive for prostate cancer on cognitive MP-MRI-targeted TRUS biopsy. During a systematic biopsy (irrespective of MP-MRI findings), 56% (n=37) of the study subjects were positive for prostate cancer.

Table 4 shows a significant correlation between MP-MRI prostate results and TRUS biopsy results, with a significant p-value and moderately significant kappa agreement value. A diagnostic test for the detection of prostate cancer, considering a PIRADS score of more than 3 as significant, MP-MRI has 72.22% specificity, 87.5% sensitivity, 89.36% PPV, and 68.42% NPV with an overall diagnostic accuracy of 83.33%. Correlation between MP-MRI-targeted biopsy and systematic biopsy, with final TRUS biopsy results, both showing a significant p-value and moderate level of agreement measured on inter-rater kappa agreement, suggesting that both types of biopsy schemes are statistically significant.

**Table 4:** Inter-rater agreement between diagnostic modalities and transrectal ultrasound biopsy MP-MRI: multi-parametric MRI; TRUS: transrectal ultrasound

Diagnostic Method	Result	TRUS Biopsy: Benign (n=18)	TRUS Biopsy: Malignant (n=48)	Total	p-value	Kappa
MP-MRI Result	Benign	13 (19.70%)	6 (9.09%)	19 (28.79%)	<0.0001	0.587
	Malignant	5 (7.58%)	42 (63.64%)	47 (71.21%)		
	Total	18 (27.27%)	48 (72.73%)	66 (100.00%)		
Cognitive Targeted Biopsy	No	13 (19.70%)	9 (13.64%)	22 (33.33%)	<0.0001	0.500
	Yes	5 (7.58%)	39 (59.09%)	44 (66.67%)		
	Total	18 (27.27%)	48 (72.73%)	66 (100.00%)		
Systematic Biopsy	No	18 (27.27%)	11 (16.67%)	29 (43.94%)	<0.0001	0.647
	Yes	0 (0.00%)	37 (56.06%)	37 (56.06%)		
	Total	18 (27.27%)	48 (72.73%)	66 (100.00%)		

Table 5 shows a comparison between the sensitivity, PPV, specificity, and NPV of MP-MRI as a diagnostic test for the detection of prostate cancer. Taking PIRADS 3 as a significant value for prostate cancer detection increases the sensitivity further, but the specificity is severely compromised, which shows that further evaluation of PIRADS 3 lesions is needed for better estimation of the incidence of malignancy. The PIRADS 5 lesion in the present study shows a specificity and PPV of 100%.

**Table 5:** Comparison of sensitivity, specificity, PPV, and NPV of MP-MRI taking PIRADS 3 or more and PIRADS 4 or more as significant values for the detection of prostate cancer MP-MRI: multi-parametric MRI; PPV: positive predictive value; NPV: negative predictive value; PIRADS: Prostate Imaging Reporting and Data System

Criterion	Sensitivity	95% CI	Specificity	95% CI	PPV	95% CI	NPV	95% CI
≥3	97.92	88.9 - 99.9	11.11	1.4 - 34.7	74.6	62.1 - 84.7	66.7	9.4 - 99.2
≥4	87.5	74.8 - 95.3	72.22	46.5 - 90.3	89.4	76.9 - 96.5	68.4	43.4 - 87.4
5	29.17	17.0-44.1	100	81.5-100	100	76.8-100	34.6	22.0-49.1

Table 6 shows a comparison of MP-MRI, cognitive MP-MRI-targeted TRUS biopsy alone, systematic biopsy alone, and cognitive MP-MRI-targeted with concurrent systematic biopsy results, respectively (taking cognitive targeted + systematic biopsy as the gold standard). The results show that the MP-MRI has a decent diagnostic accuracy, which can be utilized in the decision-making of patients who should be subjected to a TRUS biopsy. Cognitive-targeted biopsy results are almost comparable in our study, and the addition of systematic biopsy with cognitive-targeted biopsy gives very high diagnostic accuracy. High sensitivity (91%) and negative predictive value (84%), with a good diagnostic accuracy of 72.72%, are of utmost clinical importance and make MP-MRI an ideal test for screening clinically significant prostate cancer.

**Table 6:** Comparison of MP-MRI, cognitive targeted biopsy, systematic biopsy, and concurrent cognitive +systematic biopsy as a diagnostic test for the detection of prostate cancer taking concurrent cognitive + systematic biopsy as the gold standard MP-MRI: multi-parametric MRI; AUC: area under the curve

	MP-MRI	Cognitive biopsy results	Systematic biopsy results	Concurrent cognitive + systemic biopsy results
Sensitivity (95% CI)	87.5% (74.75% to 95.27%)	81.25% (67.37% to 91.05%)	77.08% (62.69% to 87.97%)	91.25% (82.60% to 95.60%)
Specificity (95% CI)	72.22% (46.52% to 90.31%)	72.22% (46.52% to 90.31%)	71.20% (67.47% to 84.07%)	77.02% (46.52% to 90.31%)
AUC (95% CI)	0.8% (0.68% to 0.89%)	0.77% (0.65%Z to 0.86%)	0.72% (0.68% to 0.85%)	0.86 (0.75% to 0.93%)
Positive Predictive Value (95% CI)	89.36 % (76.90% to 96.54%)	88.64% (75.44% to 96.21%)	83.5% (76.51% to 92.60%)	90.57% (79.34% to 96.87%)
Negative Predictive Value (95% CI)	68.42% (43.45% to 9 6.45%)	59.09% (36.35% to 79.29%)	62.07% (42.26% to 79.31%)	88.36% (75.29% to 98.30%)
Diagnostic Accuracy	83.33%	78.79%	74.62%	92.42%

## Discussion

The study subjects under investigation were in the range of 49-78 years of age, and the mean age of the study subjects was 66.82 ± 6.9 years. Of these, 50% of the subjects were in the sixth decade, followed by the seventh decade. S. PSA levels were in the range of 4.98-83 ng/ml with a mean of 18.7±14.51 and a median value of 15.1. In our study group, a cut-off value for S. PSA value of more than 4 ng/ml was taken as the cut-off for further evaluation of prostate cancer.

Mistry K et al., in 2003, conducted a meta-analysis of 13 studies, in which a PSA value of more than 4 ng/ml was taken as the cut-off and detected to have sensitivity, specificity, and PPV of 72.1%, 93.2%, and 25.1%, correspondingly, for prostate cancer [7]. In our study, the MP-MRI prostate of the subjects showed DWI changes in 95.45% and ADC in 69.70%. T2-weighted imaging revealed changes in 51.5% of the study subjects in the transition zone and approximately 93% of the study subjects in the peripheral zone.

Our investigation is similar to a study by Haider MA et al., which showed that T2-weighted imaging alone was not as effective as T2WI in addition to DWI in detecting and localizing prostate cancer [8]. MP-MRI prostate in our study was detected to have 87.5% sensitivity, 72.22% specificity, 89.36% PPV, 68.42% NPV, and a diagnostic accuracy of 83.33%, keeping the PIRADS score of 4 or more as significant. Whereas for determining clinically significant prostate cancer, taking a Gleason‘s score of more than 7 as significant, MP-MRI showed a sensitivity of 91%, specificity of 51.61%, PPV of 68%, and NPV of 84%. Diagnostic accuracy for the detection of clinically significant prostate cancer has been found to be 72.72%.

Our results were similar to Drost FJH et al., 2017, who conducted a study on MRI pathways and TRUS-guided biopsy to identify clinically significant prostate cancer and found it to have a sensitivity of 91% and a specificity of 37% [9]. Cognitive-targeted biopsy results were found to have a sensitivity of 81.25%, specificity of 72.22%, PPV of 88.64%, and NPV of 59.09%, taking concurrent cognitive and systematic biopsy as the gold standard. Systematic biopsy in our study revealed a sensitivity of 77.08% and diagnostic accuracy of 74.62%, which is almost similar to cognitive-targeted biopsy. Inter-rater kappa agreement between cognitive-targeted biopsy results alone and systematic biopsy alone with TRUS-guided prostate biopsy revealed a significant association with a highly significant p-value (<0.0001) for both.

Our findings concurred with those of Siddiqui MM et al. (2013), who carried out a study whose main goal was to compare targeted and standard biopsy techniques for the detection of high-risk prostate cancers with Gleason's score (greater than or equal to 4+3) and whose secondary goal was identifying low-risk prostate cancers (Gleason's 3+3 or low volume 3+4) [10]. In comparison to ultrasound-guided biopsy, research found that MR and ultrasound-guided targeted biopsy identified 17% fewer low-risk tumors and 30% more high-risk instances.

Our results were also similar to Pinto PA et al., who concluded that prostate biopsy employing MRI/ultrasound fusion corresponds with multiparametric magnetic resonance imaging and enhances cancer detection after TRUS biopsy [11]. The incidence of cancer found in patients was statistically correlated with the level of suspicion on MRI, and MRI/US fusion-guided biopsy found more cancers than standard 12-core biopsy alone.

Our results were consistent with Tonttila PP et al., who concluded that while MP-MRI-targeted biopsy improves the detection of clinically significant prostate cancer, systematic TRUS-guided biopsy remains essential to avoid missing important cases [12]. In our study, combining cognitive MP-MRI-targeted biopsy with systematic biopsy significantly improved diagnostic accuracy.

In our study, MP-MRI cognitive-targeted TRUS biopsy, along with concurrent systematic biopsy, increases the prostate cancer detection rate in comparison to either of the modalities used alone. A total of 48 cases were found to be positive for carcinoma prostate, out of which 44 cases were positive in cognitive targeted TRUS biopsy. Six percent (6%) of the cases (4 cases) were detected only with systematic biopsy, and corresponding changes in MP-MRI in these cases were not detected. Our results were similar to Sathianathen NJ et al., 2019, which concluded that more than 1 in 10 patients would have significant disease if a concurrent systematic biopsy was not performed on men undergoing TRUS/MRI fusion-guided biopsy [13]. This finding proposed a clinical prediction model that might be utilized for improving patient selection for concurrent systematic biopsy to reduce the number of significant cancers that are missed.

Our results were in contrast to a study by Yarlagadda VK et al., which concluded that although the aforementioned research primarily considers high-grade disease, MRI/TRUS fusion-guided biopsy enables comparable cancer diagnosis with much fewer needle cores in biopsy-naïve men [14].

Limitations

While this study provides valuable prospective data on the correlation between PIRADS scoring and TRUS-guided biopsy results in an Indian cohort, its limitations include single-center sampling, modest patient numbers, potential observer bias, lack of long-term follow-up, and absence of multivariable/health-economics analyses. Acknowledging these caveats will strengthen the discussion section and help readers interpret the findings in context.

## Conclusions

MP-MRI prostate serves a crucial part in diagnosing prostate cancer in the current era, being a more sensitive and specific noninvasive, radiation-free modality. PIRADS scoring makes this modality a uniform, reproducible, and observer-independent technique for the detection of prostate carcinoma. Cognitive MP-MRI targeted TRUS biopsy is a sensitive method for the detection of prostate carcinoma in experienced hands. PIRADS scoring of 4 or more is highly suggestive of clinically significant prostate carcinoma, as observed by a significant correlation between PIRADS scoring and Gleason‘s scoring. 

## Appendices

**Table 7:** PIRADS scoring in MP-MRI of the prostate MP-MRI: multi-parametric MRI; DWI: diffusion weighted imaging; ADC: apparent diffusion coefficient; PIRADS: Prostate Imaging Reporting and Data System Adapted by the authors from: [15]

PIRADS Score	Assessment Category	Likelihood of Clinically Significant Cancer	T2-Weighted Imaging (T2WI)	DWI/ADC	Dynamic Contrast Enhancement (DCE)
1	Very Low	Clinically significant cancer is highly unlikely	Normal	No abnormality or minimal hypointensity	No enhancement
2	Low	Clinically significant cancer is unlikely	Linear or wedge-shaped	Mild hypointensity or slightly increased diffusivity	No or minimal enhancement
3	Intermediate	Clinically significant cancer is equivocal	Heterogeneous or ill-defined	Intermediate signal intensity	Equivocal
4	High	Clinically significant cancer is likely	Focal lesion, moderately hypointense	Marked hypointensity, high DWI signal, low ADC	Early focal enhancement
5	Very High	Clinically significant cancer is highly likely	Focal lesion with extracapsular extension or invasive features	Very marked restriction, definite high DWI and low ADC	Early intense enhancement
